# ANTspin: Efficient Absolute Localization Method of RFID Tags via Spinning Antenna

**DOI:** 10.3390/s19092194

**Published:** 2019-05-12

**Authors:** Leixian Shen, Qingyun Zhang, Jiayi Pang, He Xu, Peng Li, Donghui Xue

**Affiliations:** 1 School of Computer Science, Nanjing University of Posts and Telecommunications, Nanjing 210023, China; B16041522@njupt.edu.cn (L.S.); B16041523@njupt.edu.cn (Q.Z.); B16041505@njupt.edu.cn (J.P.); lipeng@njupt.edu.cn (P.L.); 1018041229@njupt.edu.cn (D.X.); 2 Jiangsu High Technology Research Key Laboratory for Wireless Sensor Networks, Nanjing 210003, China

**Keywords:** absolute localization, RFID, spinning antenna, RSSI

## Abstract

The Global Positioning System (GPS) has been widely applied in outdoor positioning, but it cannot meet the accuracy requirements of indoor positioning. Comprising an important part of the Internet of Things perception layer, Radio Frequency Identification (RFID) plays an important role in indoor positioning. We propose a novel localization scheme aiming at the defects of existing RFID localization technology in localization accuracy and deployment cost, called ANTspin: Efficient Absolute Localization Method of RFID Tags via Spinning Antenna, which introduces a rotary table in the experiment. The reader antenna is fixed on the rotary table to continuously collect dynamic data. When compared with static acquisition, there is more information for localization. After that, the relative incident angle and distance between tags and the antenna can be analyzed for localization with characteristics of Received Signal Strength Indication (RSSI) data. We implement ANTspin using COTS RFID devices and the experimental results show that it achieves a mean accuracy of 9.34 cm in 2D and mean accuracy of 13.01 cm in three-dimensions (3D) with high efficiency and low deployment cost.

## 1. Introduction

At present, Global Positioning System (GPS) has achieved fruitful results in outdoor positioning, which can achieve high localization accuracy and it is widely applied in map navigation [1]. However, GPS cannot capture accurate information in indoor environments, and it is difficult to meet the accuracy requirements of indoor positioning. In actual production life, there are many indoor positioning requirements in indoor navigation, building buildings, warehouse management, parking guidance, etc. Therefore, research regarding indoor positioning technology is very necessary. However, indoor positioning is more complicated than outdoor positioning with more influencing factors of the signal. Once the error is large, the whole localization system will lose its practical significance and application value, as the precision requirement is higher [2].

At present, technologies that are applied to indoor positioning mainly include Wi-Fi, Bluetooth, infrared, Radio Frequency Identification (RFID), ZigBee, and ultrasonic [3,4,5,6,7]. Commercial technologies basically adopt wireless communication base station schemes, such as RFID and Wi-Fi. RFID localization technology exchanges data through non-contact two-way communication for the purpose of mobile device identification and positioning. The current use of RFID technology to build indoor positioning systems also has great advantages. With the growing of RFID hardware and software technology, the production cost of RFID tags has been reduced to a few cents and the anti-collision algorithm for multi-tag identification is becoming more and more mature. The performance of RFID readers is also superior. It can quickly read a large number of tags in a few milliseconds, which is extremely efficient [8].

RFID has a great development space in indoor positioning as an important key technology for implementing Internet of Things. RFID indoor positioning technology has experienced more than a decade of development, bringing about a variety of localization systems. However, there is still no universally recognized stable high-precision indoor positioning system in the current research process due to some problems in these localization systems. We summarize some of the disadvantages of current localization scheme, as follows [9,10,11,12,13,14,15,16]. First, most of them collect data from a number of static antennas in fixed positions, which results in the high deployment cost in antennas. Second, when deploy the antenna in a static state, the antennas are in the edge of localization area with spatial limitation. Third, in the static identification environment, the relative positions of the antenna and tags are not changed. Due to the multipath effect and the mutual interference between tags, the tags of some specific dead angles fail to obtain enough signal energy to activate itself for feedback, which results in a “tag missing” phenomenon. Fourth, many schemes require a large number of reference tags. The cost and difficulty of deployment are simultaneously increased. Fifth, some schemes use the rotating table, place the antenna on the edge and collect data during the rotation process. Indeed, this saves antenna costs, but it requires a relatively large rotating table, which is inconvenient to deploy. In addition, localization accuracy and stability are still the most important. Although there are many methods that have achieved high accuracy, it is difficult to balance accuracy, stability, and deployment cost.

In this paper, we proposed ANTspin: Efficient Absolute Localization Method of RFID Tags via the Spinning Antenna, which introduces a rotating table in the experiment. We place the antenna in the center of rotating table, and rotate the antenna by 360 degrees at a fixed point to collect dynamic data. When compared to static schemes, more information is collected for localization. The antenna is not placed on the edge of the rotating table, so there is no need for large rotating table, and the scanning area during the rotation of the antenna is all position area. ANTspin locates tags by calculating the relative incident angle and distance between the antenna and tags. The path loss model can be used to calculate the distance. According to our experiments, during the rotation of the antenna, Received Signal Strength Indication (RSSI) increases first and then decreases and, the signal value is the largest when the antenna is facing the tag in a straight direction. The relative incident angle can be calculated in combination with start and end timestamp of rotation when RSSI reaches the maximum value.

The rest of paper is organized, as follows. In Section 2, a review of related research on RFID localization is provided. The RFID signal characteristics are analyzed in Section 3. In Section 4, we discussed the initial attempts of some localization methods. In Section 5, we describe ANTspin in details. Experiments and evaluation are illustrated in Section 6. Finally, we conclude this paper.

## 2. Related Research

RFID localization can be divided into relative position localization, two-dimensional (2D) plane localization, and three-dimensional (3D) space localization. In this section, we present a review of related research [17].

Relative Localization: Shangguan et al. [18] proposed the first study of relative localization, called ‘Spatial-Temporal Phase Profiling-Based Method for Relative RFID Tag Localization (STPP)’, based on the Otrack [19]. The ordering accuracy for misplaced books is about 84% and 95% for baggage handling. Wang et al. [20] propose a relative localization approach, called ‘Human Movement based Relative Localization (HMRL)’, which utilizes signal changes that are caused by arbitrary movement of human beings around tags. The system is efficient and convenient to deploy and the accuracy is 90.24%~93.11%. Based on research regarding STPP and HMRL, we propose PRDL: Relative Localization Method of RFID Tags via Phase and RSSI Based on Deep Learning [21]. By using deep learning, the variation characteristics of the RFID phase and RSSI are extracted with limited data accuracy conditions. The accuracy is as high as 97.3% in the case of high-density alignment tags at 1 cm.

2D Localization: The mature localization algorithms in RFID 2D localization mainly include Landmarc, Bvire, Nvire, and Vire [9,10], and they laid the foundation for RFID localization. Meanwhile, localization methods and algorithms that are based on Angle of Arrival (AOA), Time Different of Arrival (TDOA), Time of Arrival (TOA), and phase [11,22,23,24,25,26] made significant contribution to the improvement of localization accuracy. Xu et al. [12] improved the Landmarc with Bayesian probability and K-Nearest Neighbor, and the localization accuracy is about 15 cm. Fu et al. [27] proposed a scheme for the localization of a moving object based on phase and laserclustering, and the localization accuracy is about 25 cm; Vighnesh Gharat et al. [28] present an indoor localization system that is based on LF RFID that is implemented using off-the-shelf components, which achieves a mean localization error of 1.53 m with a standard deviation of 0.91 m for 352 position estimations, while keeping the localization error below 2.82 m for 90% of the cases. Yang et al. [23] presented Tagoram: Real-Time Tracking of Mobile RFID Tags to High Precision Using COTS Devices, which improves the localization accuracy to the millimeter level.

3D Localization: The scheme that is proposed by Lanxin Qiu et al. [29] leverages the spatial domain phase difference to estimate the height of objects that are inspired by the phase-based Interferometric Synthetic Aperture Radar (InSAR) height determination theory. Their experiments demonstrate a spatial median error of 0.24 m. Almaaitah Abdallah et al.[13] introduce two novel methods for 3D localization one is called Adaptive Power Multilateration (APM), which uses four RFID readers, with the localization error of 0.32 m³. The other is Adaptive Power with Antenna Array (APAA), a single RFID reader that is equipped with horizontal and vertical smart antennas, with the localization error of 0.48 m³. Jullawadee Maneesilp et al. [14] avoid the need of distance estimation according to received wireless signal strength or phase difference and achieve the localization error of 0.07 ft. Duan et al. [30] put forward an advanced method, called Tagspin, to locate the RFID reader using COTS tags, and the average accuracy is about 7.3 cm; Elma Zanaj et al. [15] focus on RFID tags localization by only using mobile readers without the inclusion of static readers, which can avoid the pre-deploying of static readers. Ferdews Tlili et al. [31] propose an approach that is based on MDS for 3D localization using active RFID tags in indoor environments.

## 3. RFID Signal Characteristic

This section focuses on the basic principles of RFID and signal characteristics.

A complete RFID system consists of a reader, an antenna, and RFID tags. The reader antenna emits a specific frequency of radio wave energy and forms a magnetic field in space. After the tag enters the magnetic field, it receives the RF signal from the reader antenna, sends out the information (passive tag) that is stored in the chip by the energy obtained, or actively transmits a signal of a certain frequency (active tag) by the tag. After the reader antenna reads the information that will be decoded, it will be sent to the central information system for data processing. In terms of communication and energy sensing between the RFID reader antenna and tags, it can be roughly divided into two types: inductive coupling and backscatter coupling. Generally, the low-frequency RFID mostly adopts the first type and the higher frequency mostly adopts the second one.

● RSSI

RSSI is an important part of RFID data used to determine the quality of links. The values are all negative and the maximum value of 0 can only be obtained under ideal conditions (such as in an anechoic chamber). This means that the tag receives all the energy emitted by the reader antenna. Equation (1) shows the indoor path loss of RSSI [12,21].

(1) $$PL(d)(dB)=PL(d_{0})+10n\mathrm{lg}\left(\frac{d}{d_{0}}\right)+X_{\sigma }$$

where *PL*(*d*) is the path loss when the distance between transceivers is d and PL(*d*_0_) is the reference path loss, which is obtained by actual test. *n* is the path loss factor, which is determined by the experimental environment. $X_{\sigma }$ is a normal random variable with a standard deviation of σ.

● Phase

Phase is one of the basic properties of RFID signal and it describes the extent to which a received signal is offset from the transmitted signal. The phase measurement of the reader output is a periodic function and it can be expressed as [18,23,30]:

(2) $$\theta =\left(2\pi \frac{2d}{\lambda }+\theta_{T}\right)\mathrm{mod}2\pi$$

where *d* is the distance between the reader antenna and the tag. The distance that the signal is transmitted from the transmission to the accepted round-trip each time is 2*d*. *λ* is the wavelength and $\theta_{T}$ is the phase shift that is introduced by the error of the system itself and the experimental environment.

● Doppler Frequency

The antenna continuously emits waves of the same frequency. According to the Doppler effect [32], when the antenna and the tag are close to each other, the time interval between two consecutive peaks reaching the tag is continuously reduced, which results in the frequency of the wave received by the tag being higher than the frequency of the antenna. Conversely, the frequency of the wave received by the tag is lower than the frequency of the antenna when the antenna and the antenna are far apart from each other. The relationship between the frequency of the wave received by the tag and the frequency of the wave emitted by the antenna can be expressed as [33]:

(3) $$f_{R}=\left(\frac{c+v_{T}}{c+v_{A}}\right)\times f_{0}$$

where *f_R_* is the frequency of the wave received by the antenna, *f*_0_ is the frequency emitted by the antenna, and *c* is the speed of light. *V_T_* is the moving speed of the tag relative to the antenna and *V_A_* is the moving speed of the antenna relative to the tag. The Doppler frequency in RFID is the frequency difference that is received by the antenna. We assume that the tags to be located are all stationary, and then the frequency difference *f_D_* received by the antenna can be expressed as:

(4) $$f_{D}=\frac{v_{A}}{c}\times f_{0}$$

**Figure 1:**
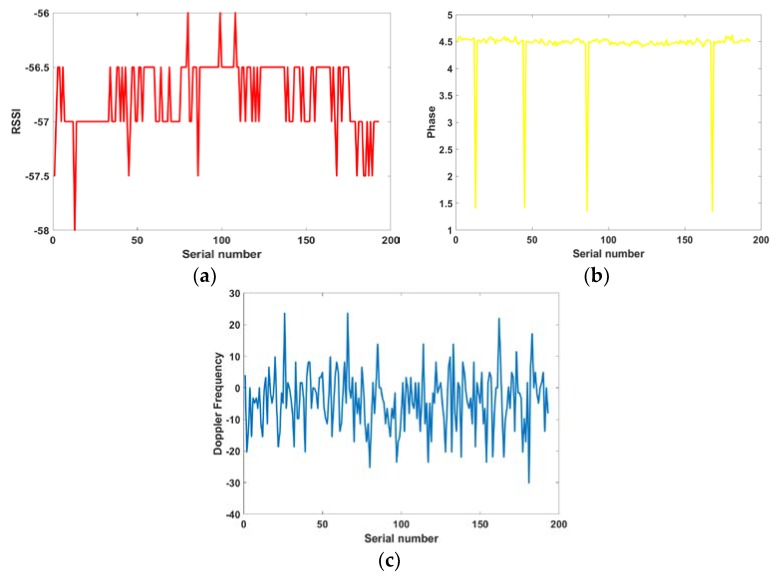
Static data fluctuation. (**a**) Received Signal Strength Indication (RSSI); (**b**) Phase; and, (**c**) Doppler Frequency.

As the experimental scene is not ideal, external RF signals and other electromagnetic signals, affect the indoor environment and there is no electromagnetic wave absorbing material in the room. It is difficult for the RF signal to conform to the law of free propagation. The multipath effect that is caused by the reflection and diffraction in the propagation process will lead to RF signal data fluctuation. Figure 1 shows the data of RSSI, phase, and Doppler frequency collected when the tag is stationary. Theoretically, the data image should be a horizontal straight line, but there is obviously varying degrees of fluctuation. The experimental results will be seriously affected if it is not processed. The processing method should be determined by different schemes and algorithms. Section 4.2 will discuss our processing method.

## 4. Initial Attempts

### 4.1. Fingerprint Library based Regional Localization

**Figure 2:**
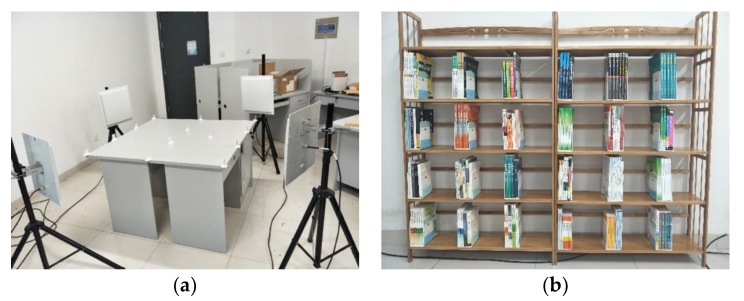
Experimental scenario. (**a**) Regional division scenario; and, (**b**) Bookshelf test scenario

The fingerprint library is one of the most commonly used methods for indoor positioning. The location fingerprint links the location in the actual environment with a certain fingerprint, and one location corresponds to a unique fingerprint. A fingerprint can be one or more characteristics of a signal. The perceived signal characteristics are then taken to match the signal characteristics in a database, which is essentially a problem regarding supervised learning. The regional localization is to divide the plane or space to be located into small pieces and then locate which region the object is in. We choose RSSI and phase as the characteristics of the signal based on the experience in PRDL [21]. In Figure 2a, the plane is divided into regions by reference tags. Place RFID antennas in four corners and move the tags multiple times to collect the location feature data. The data are prepossessed as training data and are input into the deep neural network to construct a fingerprint database. Figure 2b shows the real bookshelf test scenario [34]. The test results show that, the larger the grid of the region, the higher the localization accuracy. When the grid is 40 cm × 60 cm, the accuracy is 95.12%.

This method’s anti-interference ability is stronger, but it is more difficult to improve the accuracy and is just suitable for scenes with lower localization accuracy requirements. For example, the localization accuracy is only 60.32% when the grid is 20 cm × 30 cm. Moreover, the deployment of the system is very difficult, and the portability is weak.

### 4.2. Doppler Frequency Based Rotational Localization

**Figure 3:**
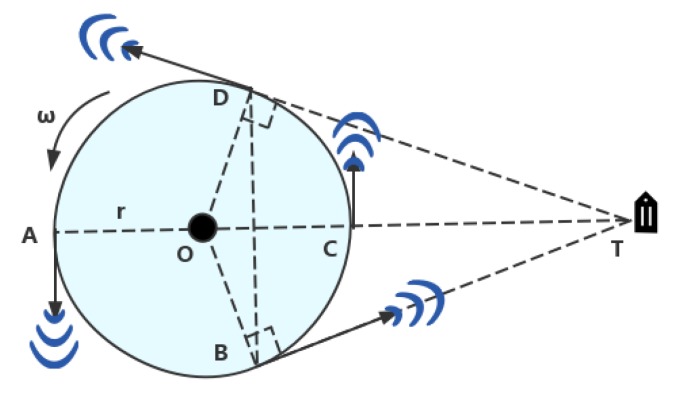
Principle analysis.

We introduced a rotating table in the experiment to rotate the antenna at the edge of the rotating table in order to reduce the cost of system deployment. Based on the Angle of Arrival (AOA) method, the transmission direction and relative incident angle of the signal between the antenna and tags are calculated using the Doppler frequency and parameters of the rotating table. As shown in Figure 3, the antenna rotates counterclockwise at an angular velocity ω at the edge of the rotating table O with a radius r, and the transmission signal collects the tag information at point T. It is easy to know that the linear velocity of the antenna rotation is *ω* × *r* from the geometric nature of the circle. When the antenna rotates from point A to point C, the relative velocity between the antenna and the tag is positive, and it reaches maximum at B (straight line BT is tangent to the circle O); when the antenna is rotated from point C to point A, the relative velocity is negative, and it is minimized at D (the line DT is tangent to the circle O). The relative velocity is 0 between two points A and C. After one rotation of the antenna, two points B and D are determined according to the change of Doppler frequency data, and two points of A and C correct the results. Finally, the tag can be located by solving the triangle BDT.

This method can reduce the cost of deployment to a large extent. However, test results are not ideal. The main reason is that the Doppler frequency is unstable. The error is very large. If the radius of the rotating table is small, it will cause a larger error. If the error of Doppler frequency can be processed well, it can be used for localization.

## 5. Materials and Methods

Based on the initial attempts, we propose ANTspin: Efficient Absolute Localization Method of RFID Tags via Spinning Antenna, which combines the advantages of previous attempts and overcomes its shortcomings. This section details the implementation of ANTspin. 

### 5.1. Data Collection

**Figure 4:**
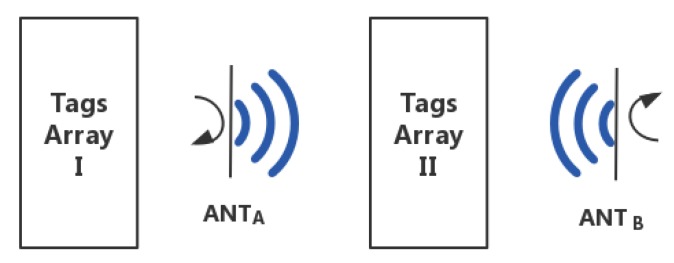
Principle analysis.

As shown in Figure 4, there are two RFID antennas, ANT_A_ and ANT_B_, and two sets of tags arrays to be located, Tags Array I and Tags Array II. If the tags array is two-dimensional, then rotate the ANT_A_ by 360 degrees to locate both Tags Array I and Tags Array II; if the tags array is three-dimensional, then rotate the ANT_A_ and ANT_B_ by 180 degrees, respectively, to locate the Tags Array II.

**Figure 5:**
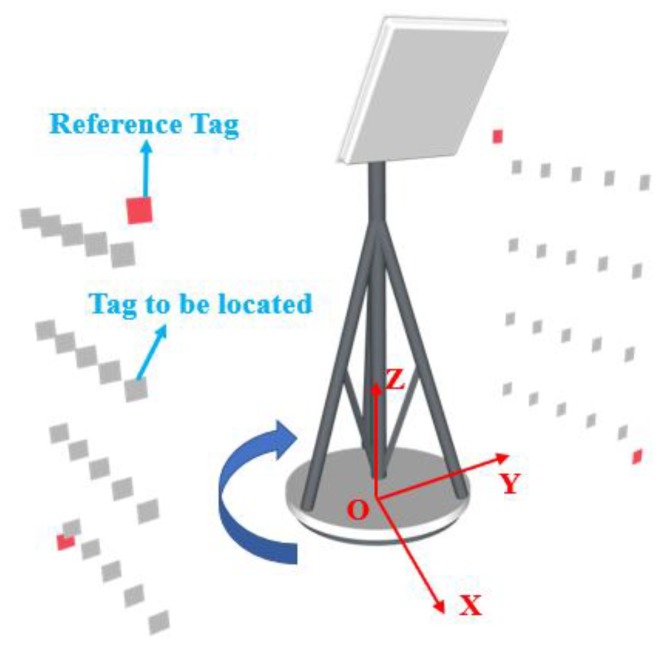
Two-dimensional (2D) deployment.

As shown in Figure 5, the antenna is placed between the tags arrays to be positioned, with the intersection of the antenna bracket and the ground as the origin O, the direction parallel to the tags array is X axis, the direction perpendicular to the tags array is Y axis, and the direction perpendicular to the ground is Z axis. The height of the antenna is H. A small number of reference tags are placed at two known coordinates of each tags array, as shown by the red tag in the figure, and the other tags on the plane are to be located. Rotate the antenna by 360 degrees from the positive direction of the X-axis. The algorithm can directly process the collected data to locate all of the tags. If the antenna rotates in the middle of the tag array, it is likely to cause symmetry in the data. The tag data of the upper and lower parts will be very similar, and it would be difficult to differ the tag in different layer. Accordingly, the antenna is placed above all of the tags to make sure that the difference in tags between different layers is obvious.

**Figure 6:**
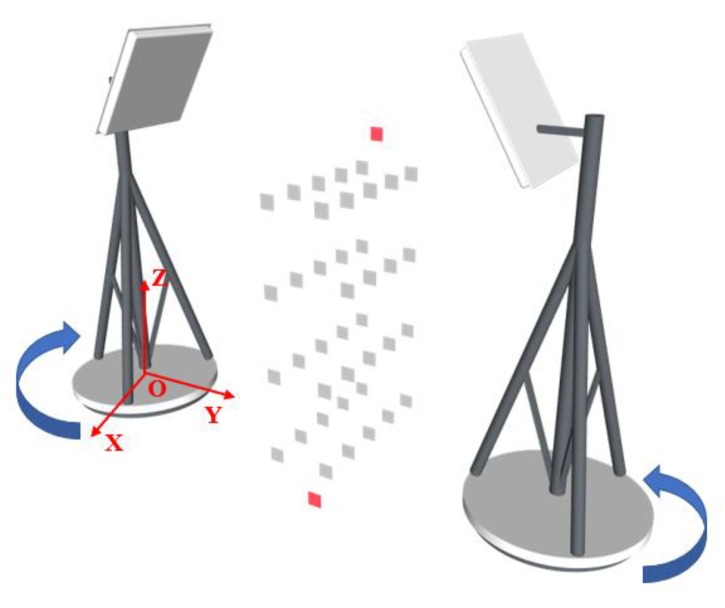
Three-dimensional (3D) deployment.

Three-dimensional localization has one more dimension on the basis of two-dimensional localization. That is, Y-axis (the Y-axis coordinate is known in two-dimensional localization). In theory, as long as the useful localization information is added, the dimension can be located. The method that we choose is to rotate the antenna by 180 data in at two known coordinates to collect data. As shown in the Figure 6, the two antennas are, respectively, located on both sides of the tags array. Of course, it is also feasible to separately place the antennas at two known positions and rotate at the same time. We recommend the former, because the antennas also interact with each other when collecting data.

### 5.2. Data Processing

**Figure 7:**
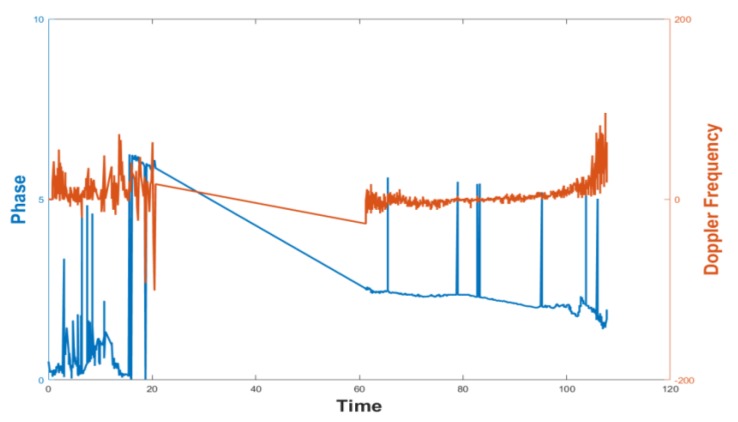
Data of Phase and Doppler Frequency.

**Figure 8:**
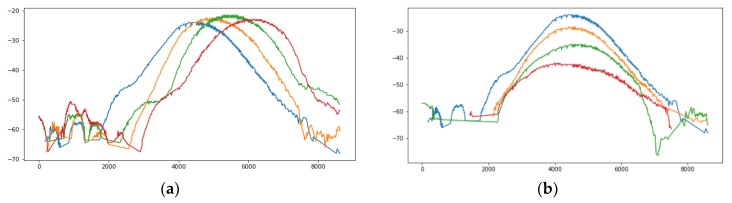
RSSI data changes. (**a**) In one row; and, (**b**) in one column.

The collected data are dirty due to the effects of multipath effects and the like. Figure 7 shows the Phase and Doppler Frequency data that were collected. It is obvious that the data are messy and it is impossible to mine effective information. Figure 8 shows the collected RSSI data and one color represents a tag. RSSI of four tags in the same column changes, as: (a), the closer the tag is to the antenna, the stronger the signal; four tags in the same row change as (b), and the tags sequentially collect the RSSI data during the rotation of the antenna. The graphics level is relatively clear, so RSSI data is used to further mine effective information.

**Figure 9:**
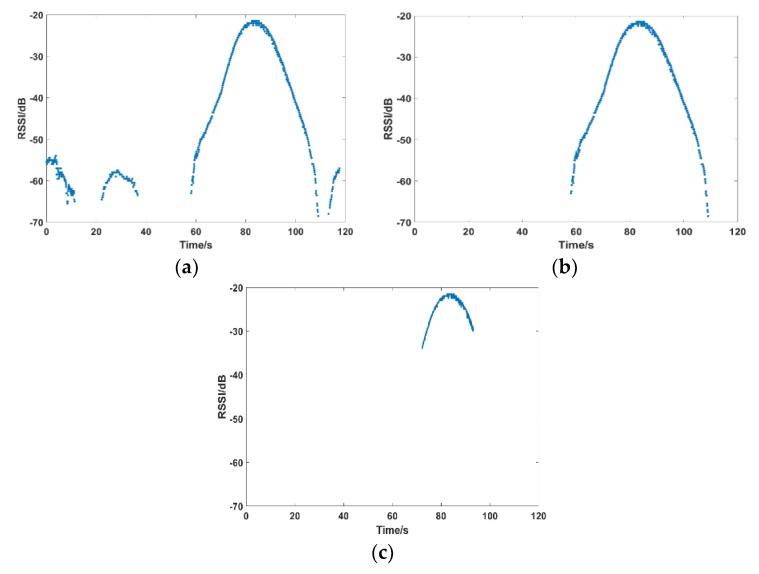
Data segmentation and interception. (**a**) Raw data; (**b**) After segmentation; and, (**c**) After interception.

Figure 9a shows the RSSI data of a tag collected. Obviously, there are interference data on the left and right. Reflection generates most of them. The middle peak is valid data. The timestamp between the interference data and valid data is mostly discontinuous. According to this characteristic, the obvious interference data is first eliminated, and the valid data of the middle large block is extracted, as shown in Figure 9b. We need to extract the maximum value of the peak, and the data below the peak is also useless, so further intercept the data according to the rotation time on both sides of the maximum value, such as intercepting the data within the 5 s interval around the timestamp of the maximum value, as shown in Figure 9c.

**Figure 10:**
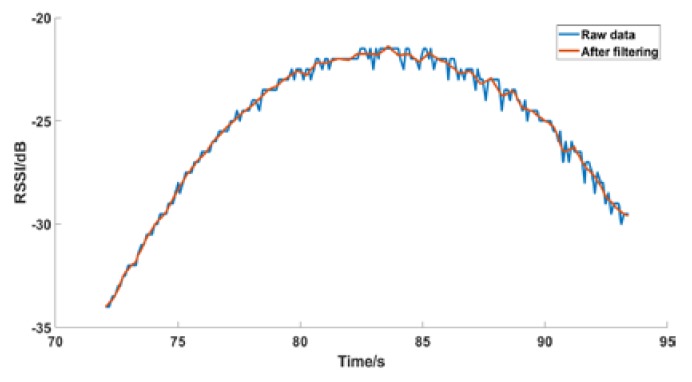
Wavelet filtering.

The obtained RSSI data are one-dimensionally discrete, and also present fluctuations. Therefore, we use a filtering algorithm to process. The commonly used filtering algorithms are Butterworth low-pass filtering, median filtering, Gaussian filtering, wavelet transform, Kalman filtering, and so on. After the experiment, the one-dimensional discrete wavelet transform is the most ideal. The wavelet is a small-area wave. It is a special waveform with limited length and an average value of 0. It retains the trend of the original data and reduces data fluctuations, as shown in Figure 10.

If we take the maximum value directly to the data sequence, it will definitely cause a large error, so we first use the quadratic function to fit the data. In formula (5), P_t_ is the fitted value, and the least squares method is used to minimize the deviation *Q*. For the unknown parameter, *y* is the actual value in Equation (6). According to the extreme value theorem, to minimize *Q*, the partial derivative must be 0, and then formula (7) can be generated.

(5) $$P_{t}=at^{2}+bt+c$$

(6) $$Q={\sum \left[y-(at^{2}+bt+c)\right]}^{2}$$

(7) $$\left\{ \begin{array}{l} \frac{\sigma Q}{a}=-2\sum (p-at^{2}-bt-c)t^{2}=0\\ \frac{\sigma Q}{b}=-2\sum (p-at^{2}-bt-c)t=0\\ \frac{\sigma Q}{c}=-2\sum (p-at^{2}-bt-c)=0 \end{array}\right.$$

**Figure 11:**
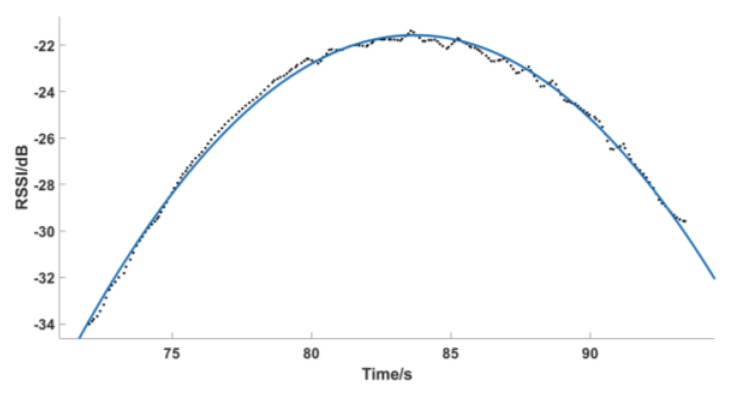
Least squares fitting.

Through the fitting curve equation, the RSSI maximum value and its corresponding timestamp can be obtained more accurately. Figure 11 shows the fitting effect.

### 5.3. Relative Incident Angle Calculation

**Figure 12:**
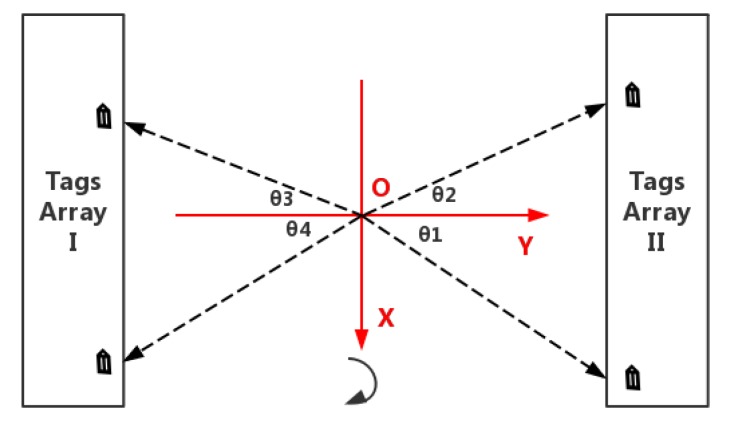
Relative incident angle analysis.

The relative incident angle is one of the necessary factors for our localization method. It is defined as the angle *θ* between the antenna direction and Y-axis. As shown in Figure 12, *θ*_1_, *θ*_2_, *θ*_3_, and *θ*_4_ are the relative incident angles of the four quadrants in the XOY plane, respectively. Note that *T_S_* and *T_E_* are the start and end time stamps during rotation, respectively. *T_Tag_* is the timestamp when the antenna direction is facing a tag in a straight direction, and the angle at which the antenna has rotated at this time ϕ is:

(8) $$\phi =2\pi \times \frac{T_{Tag}-T_{S}}{T_{E}-T_{S}}$$

In actual experiments, *T_S_* and *T_E_* acquisitions have certain errors due to various hardware delays, software delays, and artificial delays, and this will directly affect the calculation of *θ*, so we correct according to the reference tag, and record *T_R_*_1_ and *T_R_*_2_ as the theoretical timestamp and the collected timestamp when the antenna direction is facing the tag straightforward, so ϕ can be corrected to:

(9) $$\phi =2\pi \times \frac{\left(T_{Tag}-T_{S}\right)+\left(T_{R1}-T_{R2}\right)}{T_{E}-T_{S}}$$

In the process of rotating the antenna by 360 degrees, if ϕ < π, the tag is in tags array I, otherwise in tags array II. Make the following convention in order to facilitate subsequent calculation: the Y-axis coordinates of tags array I is negative. Y-axis coordinates of the tags array II are positive. *θ* of the first and fourth quadrants are positive, so *θ*_1_ and *θ*_4_ are positive, and *θ* of the second and third quadrants is negative, so *θ*_2_ and *θ*_3_ are negative, then:

(10) $$\theta =\left\{ \begin{array}{l} \frac{\pi }{2}-\phi ,\phi <\pi \\ \frac{3\pi }{2}-\phi ,else \end{array}\right.$$

### 5.4. Distance Calculation

After the data are prepossessed, the RSSI maximum value of each tag and its corresponding timestamp can be obtained. Two reference tags with known absolute positions are recorded as *r*_1_ and *r*_2_, and the following three equations can be listed, according to formula (1):

(11) $$\begin{array}{l} PL(d_{r1})(dB)=10n\mathrm{lg}\left(\frac{d_{r1}}{d_{0}}\right)+PL(d_{0})+X_{\sigma }\\ PL(d_{r2})(dB)=10n\mathrm{lg}\left(\frac{d_{r2}}{d_{0}}\right)+PL(d_{0})+X_{\sigma }\\ PL(d)(dB)=10n\mathrm{lg}\left(\frac{d}{d_{0}}\right)+PL(d_{0})+X_{\sigma } \end{array}$$

where $PL(d_{0})+X_{\sigma }$ and n are related to the experimental environment and they cannot be directly calculated. First, the data of tag to be located is subtracted from the data of the two reference tags, $PL(d_{0})+X_{\sigma }$ is directly eliminated, then:

(12) $$\begin{array}{l} PL(d)(dB)-PL(d_{r1})(dB)=10n\mathrm{lg}\left(\frac{d}{d_{r1}}\right)\\ PL(d_{r1})(dB)-PL(d_{r2})(dB)=10n\mathrm{lg}\left(\frac{d_{r1}}{d_{r2}}\right) \end{array}$$

Eliminate *n* by dividing the two equations to get:

(13) $$\frac{PL(d)(dB)-PL(d_{r1})(dB)}{PL(d_{r1})(dB)-PL(d_{r2})(dB)}=\mathrm{lg}\left(\frac{d}{d_{r1}}\right)/\mathrm{lg}\left(\frac{d_{r1}}{d_{r2}}\right)$$

The left side of the equation can be simply calculated by the collected data. We can write it as α and further get:

(14) $$\begin{array}{l} \alpha =\mathrm{lg}\left(\frac{d}{d_{r1}}\right)/\mathrm{lg}\left(\frac{d_{r1}}{d_{r2}}\right)\\ {\left(\frac{d_{r1}}{d_{r2}}\right)}^{\alpha }=\left(\frac{d}{d_{r1}}\right)\\ d={\left(\frac{d_{r1}}{d_{r2}}\right)}^{\alpha }\times d_{r1} \end{array}$$

### 5.5. 2D Localization

**Figure 13:**
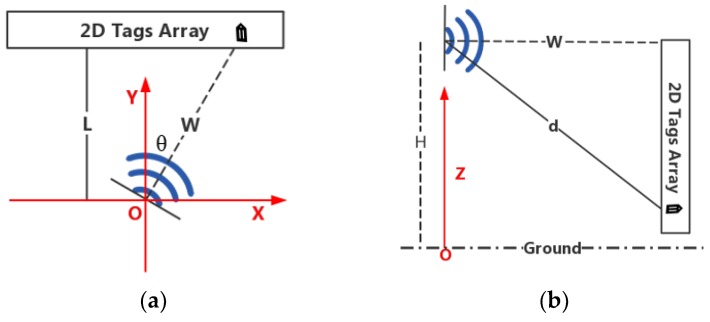
2D localization principle analysis. (**a**) Top view; (**b**) Sectional view.

For a two-dimensional scene, the tag can be located by knowing the relative incident angle and distance between one antenna and tags. Figure 13a is a top view of the system (taking one tags array as an example), the absolute position of the antenna and tags array is fixed, and the distance between them *L* is known. We record the three-dimensional coordinates of the tag as *X_T_*, *Y_T_*, and *Z_T_*. According to the method in Section 5.3, the angle *θ* in the figure can be calculated. *X_T_*, *Y_T_* of the tag are (*L* × tan *θ*, L) and the distance from the antenna to the tag in the XOY plane is *W* = |*L*/cos*θ*|. Figure 13b is a sectional view of the antenna and the tag, wherein *W* is known, the distance d from the antenna to the tag in space is calculated through the method depicted in Section 5.4. According to the Pythagorean theorem, the vertical distance between the tag and the antenna can be obtained and the antenna is placed at height of *H*, then $Z_{T}=H-\sqrt{d^{2}-W^{2}}$, so *X_T_*, *Y_T_*, *Z_T_* are :

(15) $$\left(X_{T},Y_{T},Z_{T}\right)=\left(L\times \tan\theta ,L,H-\sqrt{d^{2}-W^{2}}\right)$$

### 5.6. 3D Localization

Use an antenna to rotate by 180 degrees at a constant speed at two known coordinates. For a tag to be located, there are two sets of angles and distances information. On this basis, there are three ideas for three-dimensional localization.

1. The first type is based on the AOA algorithm. Two angular directions intersect to get the coordinate on the XOY plane, and the distance between the antenna and tags determines the Z-axis coordinate.
2. Calculate the distance between the tag and the two antennas at first. Take the antenna as the center and distance as the radius of the sphere. The intersecting loop of the two spheres determines the Y-axis coordinate. After that, the problem is converted into two-dimensional localization.
3. Third, the Y-axis coordinates are determined by using the distance data of the two antennas, and the coordinates on the XOZ plane is determined by the intersection of the two relative incident angles and the loop.

**Figure 14:**
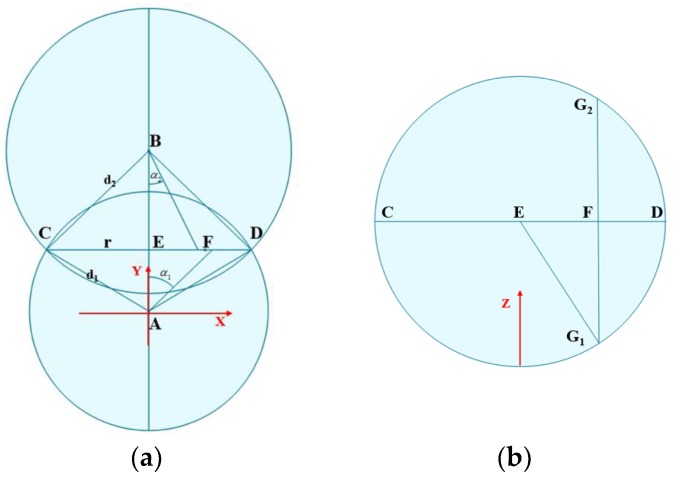
3D localization principle analysis. (**a**) Top view; and, (**b**) Sectional view.

The AOA algorithm has already been mentioned in Section 4.2 and it will not be described here. The other two methods are discussed, as follows. Figure 14a is a top view of the system deployment. Point A and B are the positions of two known coordinates where place antennas and the two antennas are, respectively, recorded as ANT_A_ and ANT_B_. The distance between the two antennas is L’. The distances d_1_ and d_2_ of the ANT_A_ and ANT_B_ to the tag to be positioned are, respectively, calculated according to the method in Section 5.4. For each antenna, the tag may be at any position on the spherical surface with the antenna position as the center of the sphere and the distance d being the radius. The height of the antenna from the ground is H. The equations of the two spheres are:

(16) $$\begin{array}{l} x^{2}+y^{2}+{\left(z-H\right)}^{2}=d_{1}{}^{2}\\ x^{2}+{\left(y-L^{\prime }\right)}^{2}+{\left(z-H\right)}^{2}=d_{2}{}^{2} \end{array}$$

The loop where the two spherical surfaces intersect is the alternative position of the tag, as shown in CD in Figure 13a,b. The loop equation is:

(17) $$\left\{ \begin{array}{l} x^{2}+{\left(z-H\right)}^{2}=d_{1}{}^{2}-y^{2}\\ y=\frac{d_{1}{}^{2}-d_{2}{}^{2}+L^{\prime }{}^{2}}{2L^{\prime }} \end{array}\right.$$

The radius of the loop is *r* and *Y*-axis coordinate has been obtained. At this time, the coordinates of *X*-axis and *Z*-axis can be directly solved into the two-dimensional localization model, as mentioned in the second method above. However, a lot of effective information is wasted; in order to make full use of the collected data and improve accuracy, we undertake further analysis, as mentioned in the third method above. *X*-axis coordinate of the tag can be determined by the relative incident angle of the antenna. The two antennas have two relative incident angles, *α*_1_ and *α*_2_, and the calculated two *X*-axis coordinates are averaged as the final result, that is Point F in Figure 13a, then:

(18) $$X_{T}=\frac{\sqrt{d_{1}{}^{2}-r^{2}}\times \tan\alpha_{1}+\sqrt{d_{2}{}^{2}-r^{2}}\times \tan\alpha_{2}}{2}$$

Substitute the *X*-axis coordinate values into the loop equation to solve two *Z*-axis coordinate values, which are shown as *G*_1_ and *G*_2_ points in Figure 13b. Previously, the tags are all below the antenna, so the *G*_1_ point was chosen to solve the coordinates of all three dimensions of the tag.

## 6. Results and Evaluation

### 6.1. Hardware Equipment and Deployment

The RFID hardware used in our experiment includes an Impinj Speedway R420 reader, a UHF antenna, and a set of H47 UHF passive tags (52 mm × 52 mm). The frequency of the signal that was emitted by the reader is between 902 MHz~928 MHz. The T360-A03 rotary table with radius of 30 cm is also equipped. The algorithms are executed on a Shinelon PC equipped with an Intel(R) Core(TM) i7-6700HQ CPU (2.60 GHz, 4 cores), a GTX1060 6G GPU, and 16 GB RAM.

**Figure 15:**
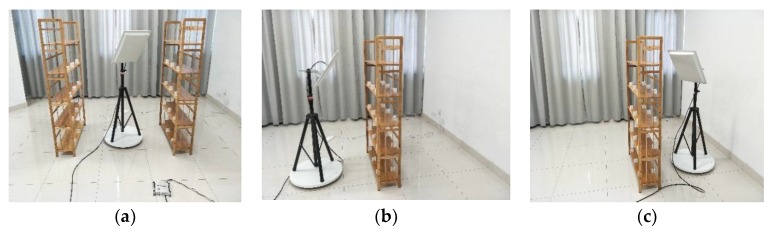
Experimental scenarios. (**a**) two-dimensional (2D); (**b**) three-dimensional (3D); and, (**c**) three-dimensional (3D).

The experimental scenario is deployed as shown in Figure 15. The antenna is mounted on a rotating table with wooden shelves on both sides, which are used to place tags. The reader and antenna are connected to the computer via Wi-Fi. The size of the bookshelf is 150 cm × 89 cm × 26 cm, the height between the layers is 30 cm, the center of the rotating table is 75 cm from the bookshelf, and the height of the center of the antenna is 130 cm. In the 3D localization scene, two antennas are placed on both sides of the three-dimensional tag sequence, and the distance between the antennas is 150 cm. The rotary table we chose is fully automatic by electronic motor. It can be rotated at a constant speed by turning on the switch. The rotation speed is 3 degree/s. During each rotation, about 500 data is collected for each tag. Both 2D and 3D are the same configuration.

### 6.2. 2D Plane Localization Accuracy

**Figure 16:**
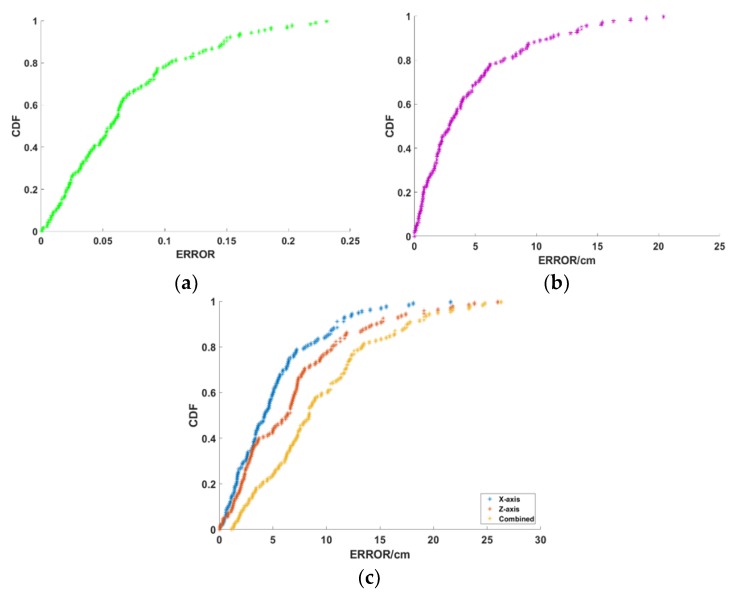
Localization error in 2D. (**a**) Relative incident angle error in 2D; (**b**) Distance calculation error in 2D; and, (**c**) Localization error in 2D.

We first validate the performance of ANTspin in a 2D plane. The scenario is deployed, as shown in Figure 15a. We move the tags to be located and then collected about 150 test cases. Figure 16 plots the CDF of localization error in 2D. The mean error of relative incident angle is 0.0674, 80% of the errors are less than 0.1 with minimal error of 0.0006 and maximal error of 0.231. The mean error of distance calculation is 4.37 cm and 90% of the errors are less than 10cm with minimal error of 0.12 cm and maximal error of 20.4 cm. Errors over 15 cm are individual phenomena. The mean distance error of ANTspin under the 2D plane scenario is 5.11 cm in the X-axis, 6.82 cm in the Y-axis, and 9.3 cm in combined dimension; 80% of the errors are less than 15 cm with minimal error of 1.16 cm and maximal error of 26.3 cm. We tested some existing schemes in a similar experimental environment, and the experimental results show that ANTspin performed better than Landmarc (33 cm), Vire (19 cm), SVR-Landmarc (23 cm), and BKNN (15 cm) [9,10,12,35].

### 6.3. 3D Space Localization Accuracy

**Figure 17:**
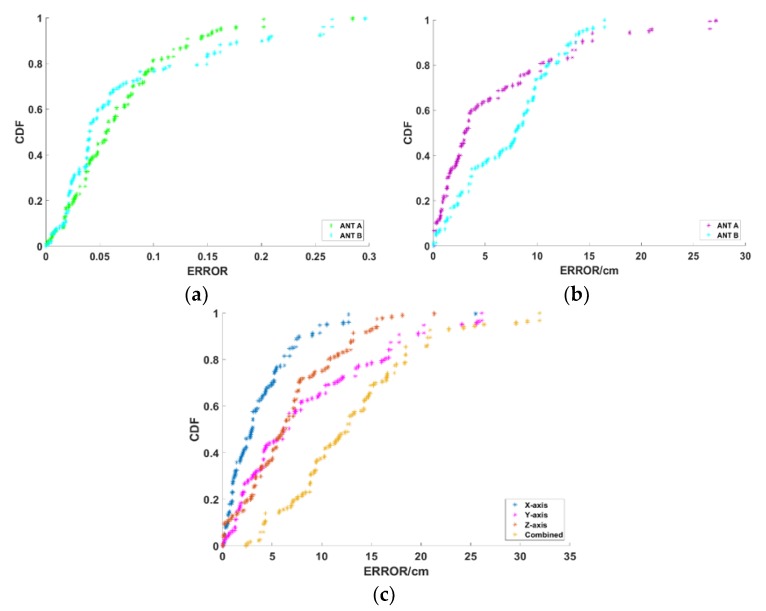
Localization error in 3D. (**a**) Relative incident angle error in 3D; (**b**) Distance calculation error in 3D; and, (**c**) Localization error in 3D.

After validating performance in 2D plane, we test ANTspin in a 3D space, as shown in Figure 15b,c. We separately test the three methods and, as we expected, the third method performs best, as it makes more use of the positioning information. Later, we mainly test the third method. We move the tags to be located and then collected about 100 test cases. Figure 17 plots the CDF of localization error in 3D. The mean error of relative incident angle is 0.07 in ANT_A_, 0.0733 in ANT_B_, 80% of the errors are less than 0.10 with minimal error of 0.0006 in ANT_A_, 0.0025 in ANT_B_ and maximal error of 0.28 in ANT_A_, 0.29 in ANT_B_. The mean error of distance calculation is 5.79 cm in ANT_A_, 7.34 cm in ANT_B_, 90% of the errors are less than 15 cm with minimal error of 0.08 cm in ANT_A_, 0.03 cm in ANT_B_, and maximal error of 27.17 cm in ANT_A_, 16.46 cm in ANT_B_. Errors over 15 cm are also individual phenomena. The mean distance error of ANTspin under the 3D plane scenario is 3.79 cm in X-axis, 8.37 cm in Y-axis, 6.70 cm in Z-axis, and 13.00 cm in combined dimension; 90% of the errors are less than 20 cm, with a minimal error of 2.37 cm and maximal error of 31.9 cm. We tested some existing schemes in a similar experimental environment and the experimental results show that ANTspin performed better than APM (30 cm), APAA (45 cm), RW (16 cm), WPM (32 cm), and RWD (22 cm) [13,15]. Ref. [15] is indeed a good solution. When compared to Ref. [15], ANTspin requires fewer antennas and lower equipment costs. Further, ANTspin is simpler to deploy, which makes it more portable. In addition, the analysis of various parameters in ANTspin shows that it is very robust.

### 6.4. Parameter Analysis

We will analyze different experimental parameter values’ impact on the performance of ANTspin in this subsection. The following experiments were performed in the 2D plane with the control variable method. In principle, both 2D and 3D require parameter analysis. However, the core methods of 2D and 3D are the same in this paper, they are only different just in the subsequent geometric operations. We did a simple parameter analysis on 3D and found that the conclusion is basically similar to 2D, so we just introduced parameters analysis for the 2D environment in detail.

#### 6.4.1. Tag Density

**Figure 18:**
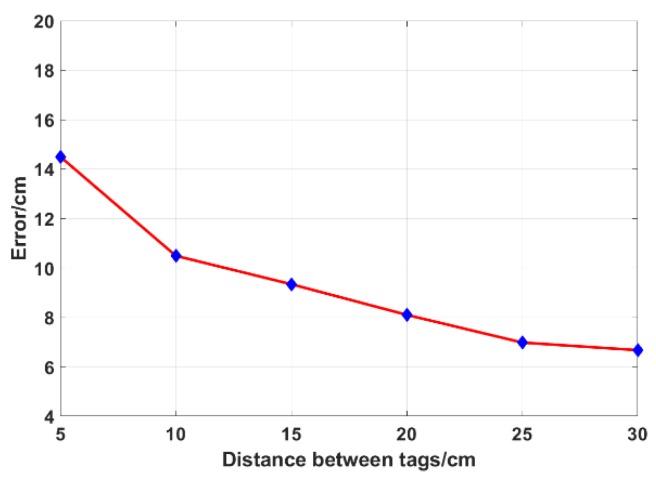
Distance between tags vs. Error.

In the RFID system, the influence between the tags cannot be ignored, so the density of the tags has a certain influence on the localization error [36,37]. We fix the antenna configuration and the plane of tags array remained unchanged, changing the space between the tags. Figure 18 shows the localization accuracy under different tag densities. The localization error is below 10 cm when the distance between tags is more than 15 cm. When it is about 25 cm or more, the influence between the tags is small, and the localization error is stable at about 7 cm. When it is 5 cm, the localization error is 14.24 cm. If the distance is smaller, the localization error is very large, and it will lose the practical significance of localization. ANTspin’s fault tolerance for tag density is sufficient for most practical applications.

#### 6.4.2. Distance between Antenna and Tag Array

**Figure 19:**
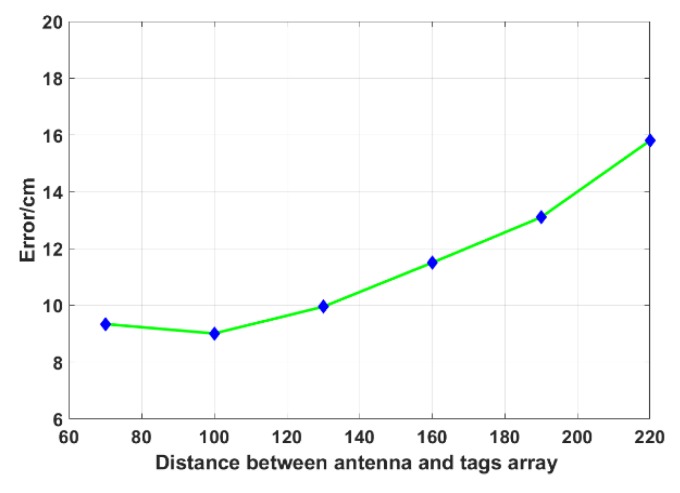
Distance between antenna and tags array vs. Error.

The configuration of our fixed tags array is unchanged, which is 20 tags with a pitch of 15 cm, in order to explore the influence of the distance between the antenna and tags array on the localization error. Increase the distance between the antenna and tags array in turn, as shown in Figure 19. The localization error is first reduced and then increased. We consider that, when the distance is small, some of the tags are at the edge of the signal coverage area, which result in low data quality, which affects the overall localization error. When the distance gradually increases, more tags enter the signal coverage area. The data quality improves, so the localization error is naturally small. In addition, as mentioned above, the error, with respect to the incident angle, directly affects the localization error, and when the distance is further increased, the influence will become larger.

#### 6.4.3. Incidence Angle of the Signal

Many studies have shown that, when the incident direction of the antenna is perpendicular to the plane of the tag, the effective aperture of the tag is the largest and the tag receives the most energy. When the incident direction is parallel to the tag plane, the energy is at its least. We place the antenna in the middle of the symmetrical tags array to explore the difference between tags. The experimental results show that the localization error of the tags placed in the middle is smaller than that at the side. During the rotation of the antenna, the angles of the signals received by the tags at the side are larger, and the energy absorption is more sufficient. Therefore, as shown in Figure 8, the peak of the RSSI curve is flat and it is difficult to distinguish the maximum value. However, the RSSI curve of the tags in the middle immediately drops after reaching the highest point. The judgment is more accurate and ultimately leads to the difference in localization error.

#### 6.4.4. Reference Tags

**Figure 20:**
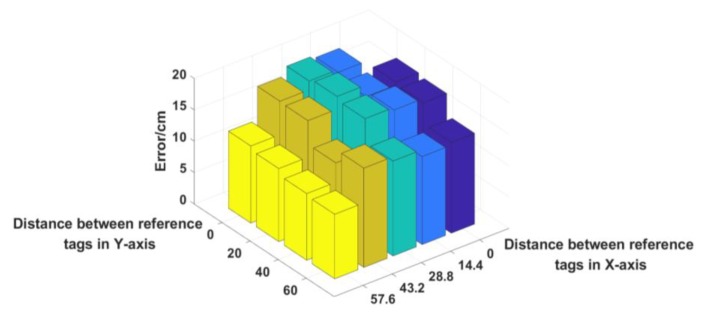
Distance between reference tags vs. Error.

Only two reference tags are involved in the experiment, and we explored all of the reference tags combinations. The result is in Figure 20, as the horizontal distance difference (X-axis) and height difference (Y-axis) between the two reference tags decreases, the error significantly increases. Therefore, the best placement of the reference tags is the diagonal corners, because the difference of data between the two reference tags is the largest, which is beneficial for subsequent calculations. Other placement methods yield large error, so simply adding reference tags will only increase the average error. Unless the reference tags are placed on all four corners, and the diagonal line is used as a group, which averages the results of the two groups and can reduce the upper limit of the positioning error to a certain extent and improve the stability of the system.

#### 6.4.5. Antenna Rotation Speed

The rotary table in the experiments is automatic. It can rotate at a constant speed by turning on the switch. There are three speeds: 3 degrees/s, 4 degrees/s, and 6 degrees/s. During each rotation, about 200–500 data are collected for each tag. It is obvious that, the slower the speed, the more complete the collected data is, which is more conducive to positioning. We tested different rotation speeds. The results show that there was no difference among them, so ANTspin can extract RSSI peaks at faster rotation speeds. As long as the antenna does not rotate at very high speed, the data it collects is basically complete, and the peak of the RSSI data is easy to extract. Accordingly, ANTspin has a large tolerance range for antenna rotation speed, which can meet the needs of most real-world applications.

#### 6.4.6. Antenna Height and Tilt Angle

The height and tilt angle of the antenna appear in the experiment as the coverage area of the signal. The natural localization error is large if the tag is at the edge of the signal coverage area. Our experiments show that ANTspin performs well in the stable and readable range of the antenna.

### 6.5. Some Discussions about ANTspin

ANTspin gets more information for localization by rotating the antenna, and it uses a small number of reference tags, which greatly reduces the deployment cost of the localization system. The time for one localization is less than two minutes, and the time of running the program is milliseconds. When compared with the traditional localization method, ANTspin expands the range of localization, but it should be noted that the actual localization range depends on the stable readable range of the antenna. For example, if the stable readable distance of the antenna is 6m, then, in the sphere space with the antenna as the center and with a radius of 6 m, the tags can be positioned more accurately. For practical applications, we use passive tags, which have limited read and write distances. They are more suitable for smart campuses, such as bookshelves and shelves, smart cities, and modern logistics applications. If active tags are used, the read/write distance is longer and the anti-interference ability is also stronger. ANTspin can be applied in a wider range of applications. 

We placed the antenna on the rotating table in the experiments, since our solution is still in the experimental stage. Integration is necessary in practical applications, which will require machinery assistance. In fact, the core idea of ANTspin is to collect dynamic data at a constant speed with the rotated antenna. Therefore, when the reader, antenna, and data acquisition module are combined together, the rotating table or mechanical device can be used to control the uniform rotation. Additional power also needs to be provided for the convenience of the equipment.

### 6.6. Application

**Figure 21:**
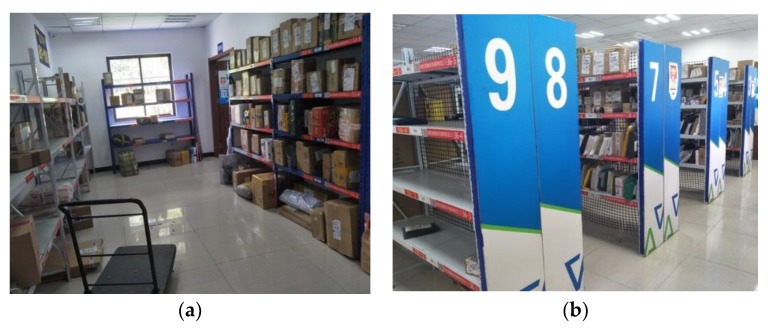
Express post stations. (**a**) Large express parcels situation; (**b**) Small express parcels situation.

We designed a modern logistics management system in [38], and located the express parcels through the fingerprint library method, as described in Section 4.1. The defect makes is obvious that the deployment is complicated. Accordingly, we can improve the positioning scheme of express parcels through ANTspin. Usually, the express parcels are divided into large and small pieces and they are placed in different places. Take a courier station as an example, as shown in Figure 21a, the shelves of large express parcels are placed around the room, which can be seen as four planes to be positioned. According to ANTspin, all of the express parcels can be located by placing a rotating antenna in the middle of the room and rotating by 360° to scan all of the shelves in the room. As shown in Figure 20b, the express parcels are placed in dense shelves to form a 3D space to be positioned. According to ANTspin, all of the express parcels can be located by placing rotating antennas on both sides of the shelves and rotating by 180° to scan the shelves. In addition, different express post stations have different layouts, which requires different deployment schemes of ANTspin in the field. After locating the express parcels, the system combines the communication technology to send the positioning information to the user via SMS, and the user can quickly find the parcel after receiving the short message.

## 7. Conclusions

Nowadays, research regarding indoor positioning is in full swing. Our proposed scheme ANTspin: Efficient Absolute Localization Method of RFID Tags via Spinning Antenna, rotate antennas to collect dynamic data, get more information for localization. The scheme can be applied in 2D and 3D localization scenes. We implement ANTspin while using COTS RFID devices and the experimental results show that it achieves a mean accuracy of 9.34cm in 2D and mean accuracy of 13.01cm in 3D. It has a good promotion prospects, with high localization accuracy, low deployment cost, and strong practical application significance.
